# Ureter mixed neuroendocrine-non-neuroendocrine neoplasm: a case report and literature review

**DOI:** 10.3389/fonc.2024.1390350

**Published:** 2024-05-31

**Authors:** Bing Zhou, Xing Gan, Xiaohua Li, Lizi Peng, Hua Hao

**Affiliations:** ^1^ Department of Pathology, Second Affiliated Hospital of Jiujiang University, Jiujiang, China; ^2^ Department of Clinical Laboratory, Wuhan Jinyintan Hospital, Wuhan, China; ^3^ Department of General Surgery, Second Affiliated Hospital of Jiujiang University, Jiujiang, China; ^4^ Department of Pathology, Jiujiang First People’s Hospital, Jiujiang, China; ^5^ Department of Pathology, Yangpu Hospital, School of Medicine, Tongji University, Shanghai, China

**Keywords:** ureter, mixed neuroendocrine-non-neuroendocrine neoplasm, MiNEN, immunohistochemistry, diagnosis

## Abstract

Cases of mixed neuroendocrine-non-neuroendocrine neoplasms (MiNENs) of the urinary system are rare, and reports of primary MiNENs in the ureter are lacking. Herein, we present the case of a 71-year-old man who presented with painless gross hematuria and weight loss. Contrast-enhanced abdominal computed tomography (CT) revealed a tumor, comprising small cell neuroendocrine carcinoma (SCNEC) and adenocarcinomatous components, attached to the ureter. The SCNEC components were strongly positive for synaptophysin, CD56 and INSM1 and adenocarcinomatous components were strongly positive for CDX2 and cytokeratin 20, respectively. Four weeks post-surgery, the patient received four cycles of cisplatin-based chemotherapy; the 7-month follow-up CT confirmed that he was healthy without disease recurrence. The occurrence of MiNEN in the ureter with SCNEC and adenocarcinomatous components is extremely rare, wherein histopathological and immunohistochemical features aid in the diagnosis MiNEN. With its aggressive nature, MiNEN can only be effectively treated by early diagnosis and radical surgery.

## Introduction

Mixed neuroendocrine-non-neuroendocrine neoplasms (MiNENs) are characterized by the presence of two major neuroendocrine and non-neuroendocrine morphological components, each accounting for >30% of the total lesion (1). MiNENs have been commonly detected in the gastrointestinal tract and recently in many other organs including the cervix, bladder, and tongue (2, 3). Urothelial carcinoma represents >90% of all malignancies in the urinary system. However, MiNEN that originate in the ureter, including neuroendocrine carcinomas (NECs) and urothelial carcinomas, are rare (4). Although the developmental mechanism closely relates to the genetic events in tumor cells, the biological behavior, prognosis, and molecular origin of MiNENs remain unclear.

Here, we report a case of a patient with a primary MiNEN of the ureter that comprised small cell neuroendocrine carcinoma (SCNEC) and adenocarcinomatous components. Additionally, we have summarized the relevant literature, to improve the medical community’s understanding of this rare type of tumor.

## Case description

**Figure 1:**
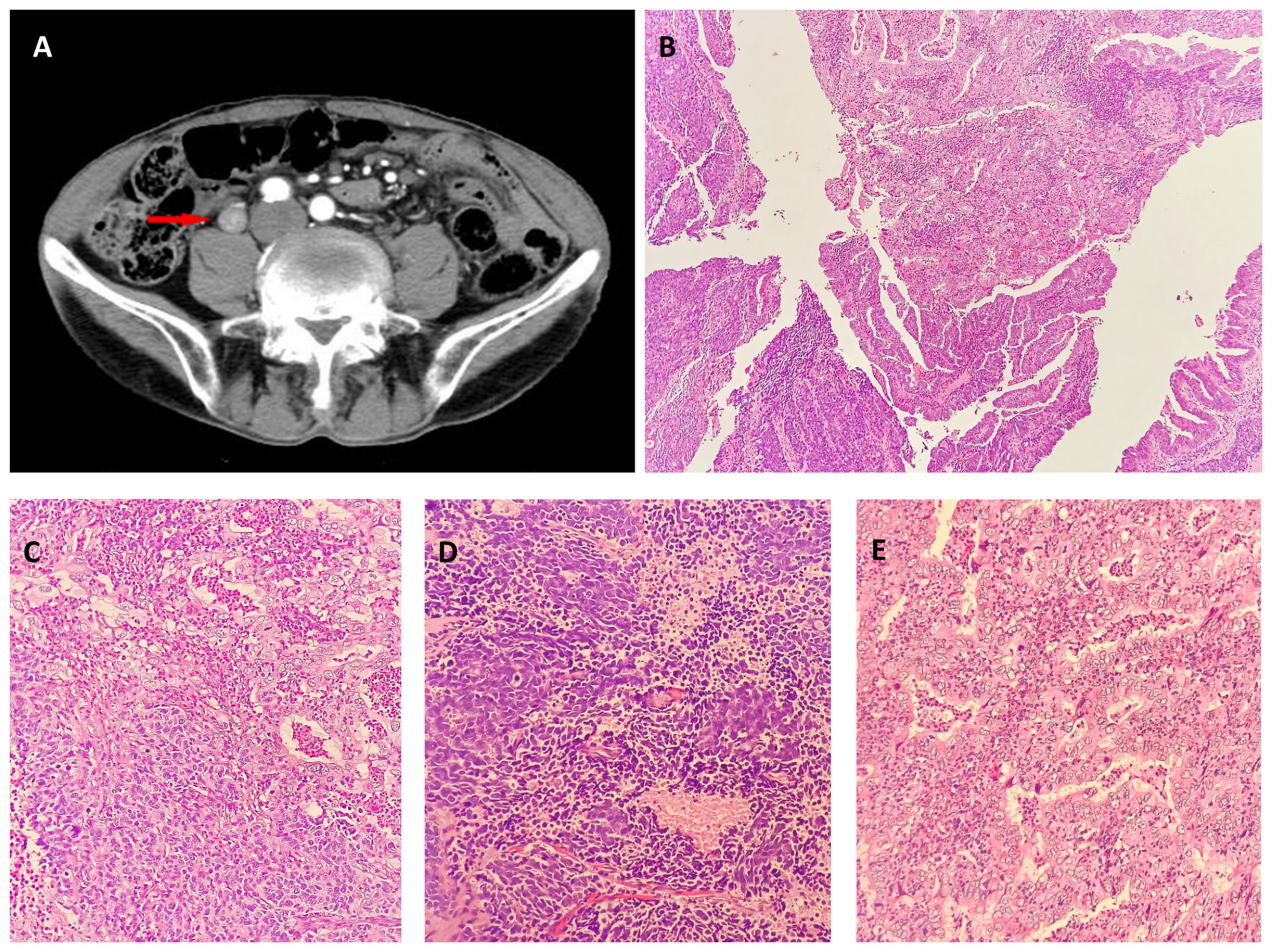
**(A)** Contrast-enhanced computerized tomography (CECT) of the left ureteral segment and left hydronephrosis. CECT revealed a 20-mm lesion on the left ureteral segment. Neuroendocrine carcinoma and adenocarcinoma in biopsy. Low (**(B)**, hematoxylin-eosin, × 40) and intermediate [**(C)**, hematoxylin-eosin, × 200] magnification showing adenocarcinoma and Neuroendocrine carcinoma. Neuroendocrine in biopsy: High magnification [**(D)**, hematoxylin-eosin, × 400]. Adenocarcinoma in biopsy: High magnification [**(E)**, hematoxylin-eosin, × 400].

A 71-year-old man presented with a 3-month history of painless gross hematuria and a 2-month history of weight loss. The patient had a 32-year and 43-year history of smoking and alcohol abuse, respectively. The patient was previously fit and had no specific life history. The physical examination findings were unremarkable. Laboratory investigations revealed that the routine blood, electrolytes, liver function, and serum amylase levels were within the reference ranges, urinalysis revealed red blood cells (3+), and negative levels of the serum tumor markers PSA, BTA and AFP. The urine cytological analysis revealed malignant epithelial neoplastic cells. Computed tomography (CT) revealed a 25-mm extensive heterogeneously-enhanced mass in the middle and lower part of his left ureteral segment and left hydronephrosis (**Figure 1A**). A subsequent ureteroscopic biopsy revealed poorly differentiated tumors. Positron emission tomography-CT and gastrointestinal endoscopy confirmed the lack of primary or metastatic lesions in other organs. The patient underwent laparoscopic left nephroureterectomy with bladder cuff resection and retroperitoneal lymph node removal. The patient has been followed up with CT postoperatively without ultrasound. Cystoscopy was normal and PET CT showed no other lesions. Postoperative laboratory indicators: all indicators are still normal, urine routine and test hematuria returned to normal. Four weeks post-discharge, the patient received four cycles of cisplatin and etoposide chemotherapy. At his 7-month follow-up, no recurrence or metastasis was noted; follow-up care is continued.

The immunohistochemical study was performed on 3-μm-thick sections using prediluted ready-to-use vials of the antibodies with an automated immunostainer.

The tissue sent for examination was a specimen of the left kidney, ureter, and part of the bladder. The ureter was 26 cm in length, and 9 cm from the opening of the ureter, a 2.5 cm × 2 cm ×1 cm mass was visible that was grayish-white in color on the section, brittle, with unclear borders, and without necrosis. Microscopically, the tumor protruding into the lumen comprised two intimately admixed morphological tumor cells (**Figures 1B, C**). Most cells (55%) exhibited solid and trabecular growth, were small, and had round or spindle-shapes, scant cytoplasm, dark staining, inconspicuous nucleoli, extensive necrosis, and a mitotic index of 60/10 high-power fields (HPFs) (**Figures 1D**). The other 45% of tumor cells exhibited glandular lumens and papillary formation, with larger tumor cells, columnar and cubic, abundant cytoplasm, conspicuous nucleoli, enteric and mucinous differentiation, and a mitotic index of 4/10 HPFs (**Figures 1E**). Conventional urothelial carcinoma components were not detected, and the tumor invaded the deep muscle. Lymph node dissection information: abdominal lymph nodes, common iliac lymph nodes, external iliac lymph nodes, obturator lymph nodes, and internal iliac lymph nodes, 0.2-1 cm in diameter. However, renal parenchymal, arteriovenous, periureteral tissue, and lymph node involvement were absent. The overall pathological stage at diagnosis was pT2N0M0.

**Figure 2:**
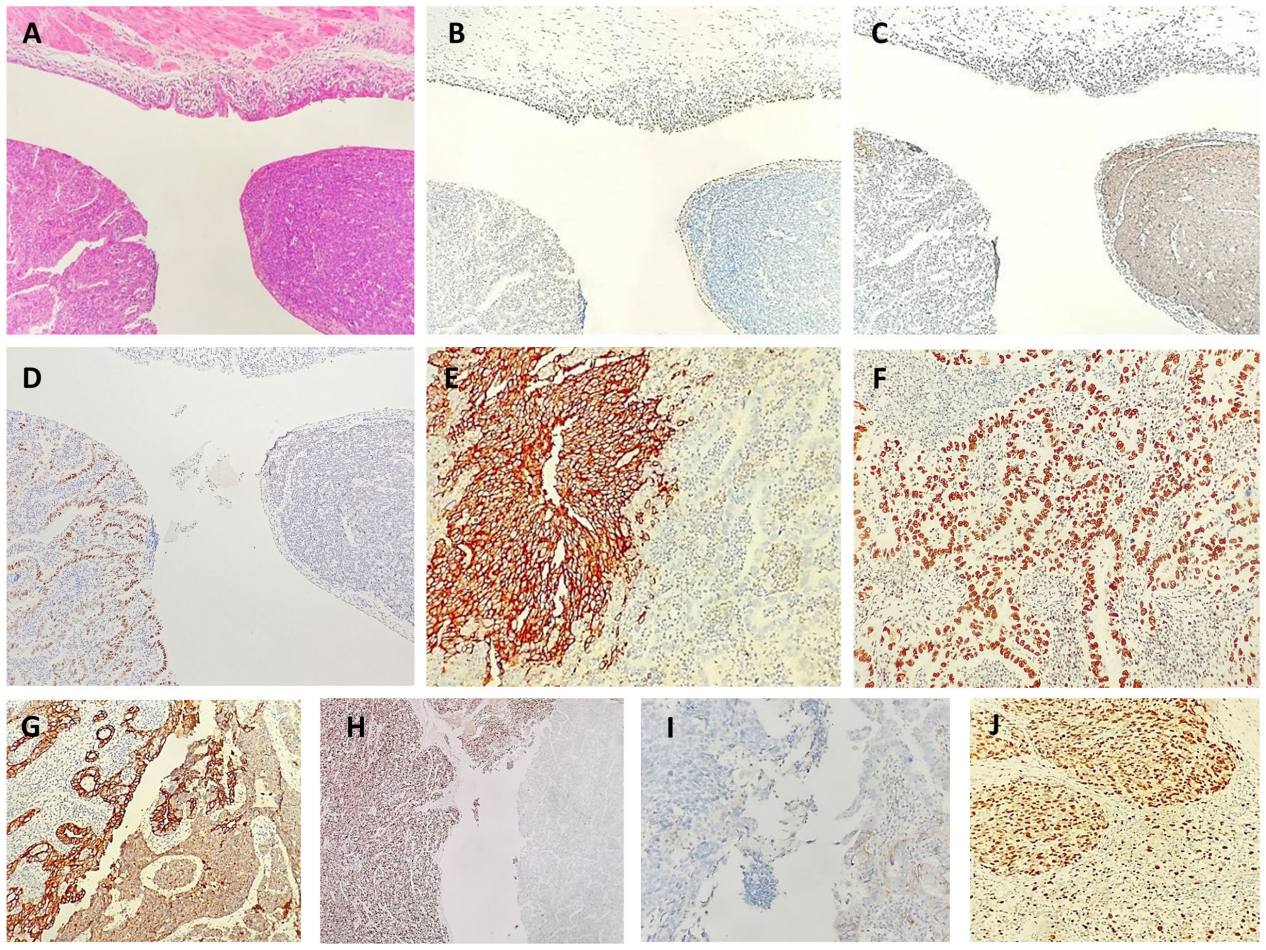
Neuroendocrine carcinoma and adenocarcinoma in biopsy. Low [**(A)**, hematoxylin-eosin, × 40]. Immunohistochemical staining showed positivity for a urothelial marker [(GATA3 **(B)**], neuroendocrine marker [synaptophysin **(C)**], adenocarcinoma marker [CDX2 **(D)**], a neuroendocrine marker [CD56 **(E)**], adenocarcinoma marker [CDX2 **(F)**, epithelial marker (CKpan **(G)**], a neuroendocrine marker [INSM1 **(H)**], a neuroendocrine marker [SSTR2 **(I)**] and proliferation index marker [Ki67 **(J)**].

Immunohistochemistry showed positive staining in different histological components (**Figures 2A–J**). The tumor cells in the solid components were positive for synaptophysin, CD56 and INSM1, partly positive for TTF-1 and negative for SSTR2. Staining with chromogranin A was very weak, and staining for CDX2, SATB2, and cytokeratin (CK)20 was negative. The tumor cells in the glandular components were positive for CDX2 and CK20 and focally positive for SATB2. The cells tested negative for synaptophysin, CD56, chromogranin A, and TTF1. Both tumor cell components were positive for CKpan, p53, MLH1, PMS2, MSH2, and MSH6 and were negative for GATA3, LCA, and HER2. The Ki-67 indices of tumor cells in the solid and glandular components were approximately 80% and 30%, respectively.

Finally, we diagnosed the patient with primary MiNEN of the ureter, with poorly differentiated neuroendocrine carcinoma (SCNEC, 55%) and moderately and highly differentiated non-neuroendocrine carcinoma (adenocarcinoma, 45%).

## Discussion

NECs of the urinary system include SCNECs, large cell neuroendocrine carcinoma (LCNEC), well-differentiated neuroendocrine tumors, and paragangliomas and account for <0.5% of all urothelial neoplasms. Over half of the primary urinary NECs are mixed with other histological components (5, 6), and more than 90% of these reports of non-neuroendocrine components were of urothelial carcinoma, followed by squamous cell carcinoma, lymphoma, sarcoma, and adenocarcinoma, in order of frequency. We summarized published cases of mixed neuroendocrine and adenocarcinomatous tumor of the urinary system between 1990 and 2023 (**Table 1**) (7–13). The male-to-female ratio was 7:1, with a median of 61 years, and it can occur in the bladder, ureter and urethra. Specifically, SCNEC is the most common NEC type, while the non-neuroendocrine components can be simple adenocarcinoma or mixed with other components. Unfortunately, the proportion of components has not been reported in most cases. In World Health Organization (WHO) classification 5th edition (2022) of urogenital tumors, mixed neuroendocrine neoplasms are still not defined as MiNEN with 30% component criteria in the manner of gastrointestinal tumors, possibly because of evidence that only a small portion of neuroendocrine carcinoma has a prognostic impact in urinary tract tumors (14). Nevertheless, in order to better understanding of this rare case of Ureter MiNEN, especially when they are morphological high-grade, more detailed clinicopathological data and percentages of each component are necessary, because they still may influence prognosis.

**Table 1:** Published cases of mixed neuroendocrine and adenocarcinomatous tumor of the urinary system.

Author	Age /sex	Race	Location	NEC type	Other contingent	Stage	Treatment	Follow-up months	Outcome
Ishikawa et al ^[7]^	53/M	Asian	Ureter	SCNEC	Ade, UC	pT4N2M0	Nephroureterectomy	8	DOD
Tsutsumi et al ^[8]^	60/M	Asian	Ureter	SCNEC	Ade, Leiomyosarcoma	pT3N0M0	Nephroureterectomy, CT	24	DOD
Pini et al ^[9]^	49/M	Non- Asian	Urinary bladder	LCNEC	Ade	pT2N0M0	Transurethral resection of the bladder	26	AWR
Raison et al ^[10]^	62/F	Non- Asian	Urethra	NA	Ade	NA	Radical urethrectomy	NA	NA
Tränkenschuh et al^[11]^	67/M	Non- Asian	Urinary bladder	SCNEC	Ade,UC	pT2bN0M0	Radical cystectomy	NA	NA
Chin et al ^[12]^	67/M	Non- Asian	Urinary bladder	NA	Ade	NA	Radical cystectomy	NA	NA
Abenoza et al ^[13]^	55/M	Non- Asian	Urinary bladder	NA	Ade	NA	Radical cystectomy, CT	30	DOD
Our case	71/M	Asian	Ureter	SCNEC	Ade	pT2N0M0	Nephroureterectomy with bladder cuff resection, CT	7	AWD

M, male; F, female; SCNEC, small cell neuroendocrine carcinoma; LCNEC, large cell neuroendocrine carcinoma; NA, data not available; Ade, adenocarcinoma; UC, urothelial carcinoma; CT, chemotherapy; DOD, dead of disease; AWR: alive with recurrence;AWD, alive without disease.

Xu et al. (15) evaluated 17 patients and reported that ureteral collision carcinoma, especially those occurring in the lower right ureter, were common in older Asian men. Concordantly, Zhong et al. (16) evaluated 32 patients with ureteral SCNECs and suggested that the cancers may be related to genetic, environmental, and lifestyle habits in Asians. This may be attributed to a higher incidence of smoking exposure, drug abuse (Chinese herbal medicine, painkillers), occupational exposure to certain aromatic amines, and lithiasis concomitant infections among Asians (4, 17). Our patient was an older Asian man with a 32-year history of smoking, consistent with the literature reports; however, the lesion was in the middle and lower left ureter, unlike previous literature reports that it is more common in the lower right ureter.

The preoperative diagnosis of ureteral MiNEN is confounded by uncharacteristic symptoms, including painless gross hematuria and a ureter mass on imaging; additionally, the accuracy of preoperative biopsy remains controversial. Therefore, final confirmation requires complete surgical excision for examination (18). Combining the clinical history and imaging findings to exclude gastrointestinal and bladder metastases is crucial for the primary diagnosis. In our case, the histomorphological features were comprehensively evaluated, and the differential diagnosis was based on immunohistochemical positivity for synaptophysin, chromogranin A, CD56, neuron-specific enolase, and characteristic markers of the corresponding non-neuroendocrine components. Furthermore, urinary cytology and biopsy pathology revealed malignancy; therefore, we completely surgically resected the tumor. Combined with the medical history, negative imaging, and spectroscopic nodes to exclude metastasis, we diagnosed the patient with primary ureteral MiNEN.

The histogenesis of the primary ureteral MiNEN remains unclear, but has been discussed extensively. At present, many scholars support the theory of MiNEN is hypothesized to originate from monoclonal pluripotent stem cells that differentiate bidirectionally when stimulated with certain factors, which could explain the mixed histological features in urogenital NECs (19). Tränkenschuh et al. (11) reported the case of a patient with primary adenoneuroendocrine carcinoma of the bladder combined with *in situ* urothelial carcinoma and observed significant similarities in the molecular features between these carcinomas, thereby highlighting their common clonal origin. Another postulated theory is that ureteral MiNEN may be caused by conversion of non-neuroendocrine to neuroendocrine components during tumor progression, including originating from residual urachus epithelium, or cystitis glandularis and intestinal anisocytosis, which would seem to explain the mix of adenocarcinoma components in MiNEN. Eckstein et al. (20) described the case of a patient with adenoneuroendocrine carcinoma of the bladder, with significant cystitis glandularis and intestinal anisocytosis surrounding the tumor. The investigators suggested that this carcinoma developed from primary adenocarcinoma of the bladder. In our case, epithelial neuroendocrine cells transformation such as glandular or intestinal epithelial chemotaxis components in the mucosa surrounding the ureteral MiNEN were not detected. In fact, almost half of urogenital NECs patients had other tumor components such as UC, squamous cell carcinoma, adenocarcinoma. Meanwhile, the conversion of non-neuroendocrine components to neuroendocrine components is rarely reported, thus further buttresses the hypothesis of the origin of multipotent stem cells.

At present, the genetic characteristics of MiNENs containing adenocarcinoma components have been investigated in the digestive system, and it is suggested that CCNE1 gain and FAT1 loss might promote the tumorigenesis of MiNENs partially through regulating cell cycle G1/S checkpoint signaling. Moreover, loss of MAPK1 and alterations in the MAPK signaling pathway, specifically occurred in the NEC component, which might contribute to the neuroendocrine differentiation (21). However, due to its rare molecular changes in the urinary system have not been reported, the relevant studies of single components can provide some references. In a report about urinary molecular typing of bladder SCNEC, a significant proportion of these tumors expressed the c-kit, TP53 and RB1 gene mutations (22, 23). Furthermore, whole exome sequencing of urinary system adenocarcinoma has found that prevalent T>A substitutions were observed among somatic mutations, and major trinucleotide contexts included 5’-CTC-3’ and 5’-CTG-3’ (24). Lu et al (25). Explore the genomic alterations of ureteral hepatoid adenocarcinoma, driver somatic mutations of TP53 and KMT2D genes were found, indicating that ureteral hepatoid adenocarcinoma has similar mutational characteristics to urothelial carcinoma. These genetic characteristics have certain potential significance for future targeted therapy and prognosis judgment, and need to be confirmed by further studies on large samples.

**Figure 3:**
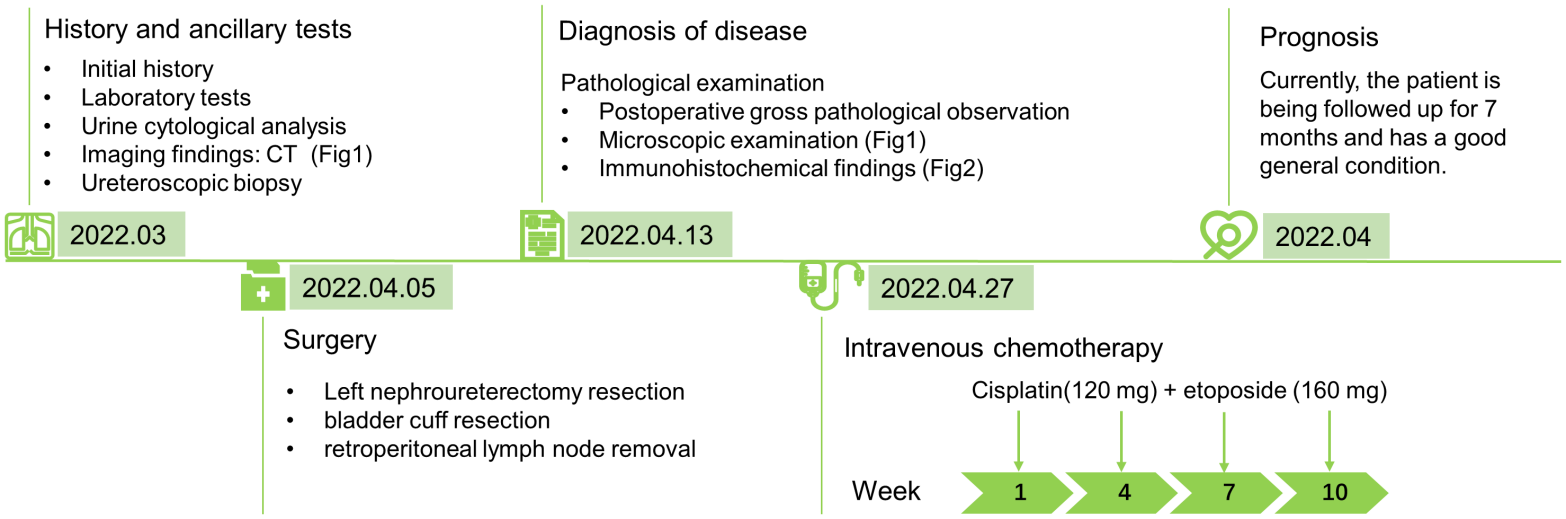
A timeline figure summarising the case diagnosis and treatment pathway.

We possess limited treatment experience owing to the rarity of ureteral MiNEN; thus, nephroureterectomy with bladder cuff resection is preferred (4, 15). Furthermore, postoperative platinum-based adjuvant chemotherapy for highly malignant SCNECs components significantly improves prognosis compared with surgical resection alone (5, 26). Qing et al. (27) attempted to treat primary ureteral SCNECs with a combination of PD-L1 ICIs and radiotherapy. Despite the unsatisfactory outcome, the report provided new insights into various treatment strategies. Furthermore, the prognosis of MiNENs is driven by their high-grade neuroendocrine components, especially in the digestive system; therefore, their prognosis is comparable with that of pure neuroendocrine cancers (28, 29). However, another large sample of urinary MiNENs was considered to be better than that of pure SCNECs (30). Pini et al. (9) reported that case of a patient with MiNEN (comprising LCNEC and adenocarcinoma) of the bladder and observed that mucinous adenocarcinoma was the most aggressive component. These findings suggest that non-neuroendocrine carcinomas are likely crucial in determining patient quality of life and prognosis. The prognostic factors of urinary MiNEN are closely related to the pathological stage; however, their association with sex remains controversial (5, 30). This disparity may be related to the rarity of the case, diverse sample sources or limited case numbers. In future work, increasing case collection and pooled case reports can make prognosis clearer. Anyhow, ureter MiNENs have a poor prognosis, which have an overall survival of approximately 15 months and a 3-year survival rate of less than 30%, with metastases occurring within 13 months in most patients. Furthermore, the most common target organs are the lungs and brain (5). In our patient, we performed a laparoscopic left nephroureterectomy with bladder cuff resection, followed by cisplatin and etoposide chemotherapy. We observed no recurrence during the 7-month follow-up. This outcome may relate to the T2 pathological stage, increased proportion of well-differentiated adenocarcinoma components, and shorter follow-up time. And a timeline figure summarizing the case diagnosis and treatment pathway was shown in **Figure 3**.

In conclusion, we report a rare case of a patient with ureteral MiNEN with SCNECs and adenocarcinoma components, whose clinical imaging presentation was mostly atypical. Characteristic histological patterns and immunohistochemical markers facilitated the accurate diagnosis. Although we lack standard MiNEN treatment options owing to its rarity, radical ureteral resection and postoperative chemotherapy can help improve prognosis, as in the cases of ureteral small cell carcinoma and adenocarcinoma.

## Data availability statement

The original contributions presented in the study are included in the article/supplementary material. Further inquiries can be directed to the corresponding author.

## Ethics statement

The studies involving humans were approved by ethics committee of Second Affiliated Hospital of Jiujiang University. The studies were conducted in accordance with the local legislation and institutional requirements. The participants provided their written informed consent to participate in this study. Written informed consent was obtained from the individual(s) for the publication of any potentially identifiable images or data included in this article.

## Author contributions

BZ: Conceptualization, Data curation, Investigation, Visualization, Writing – original draft. XG: Conceptualization, Investigation, Writing – original draft. XL: Investigation, Visualization, Writing – original draft. LP: Methodology, Writing – original draft. HH: Conceptualization, Data curation, Formal Analysis, Funding acquisition, Investigation, Project administration, Supervision, Visualization, Writing – review & editing.
